# Supplementary material for: FXR Maintains the Intestinal Barrier and Stemness by Regulating CYP11A1-Mediated Corticosterone Synthesis in Biliary Obstruction Diseases
Source: Int J Mol Sci. 2023 Aug 30;24(17):13494. doi: 10.3390/ijms241713494 (PMC10487515; doi:10.3390/ijms241713494)
Supplement: Supplementary file 1 [file ijms-24-13494-s001.zip › CKOAI190919YZ7-B-Mouse Nr1h4-AIB141-Genotyping Protocol.pdf]

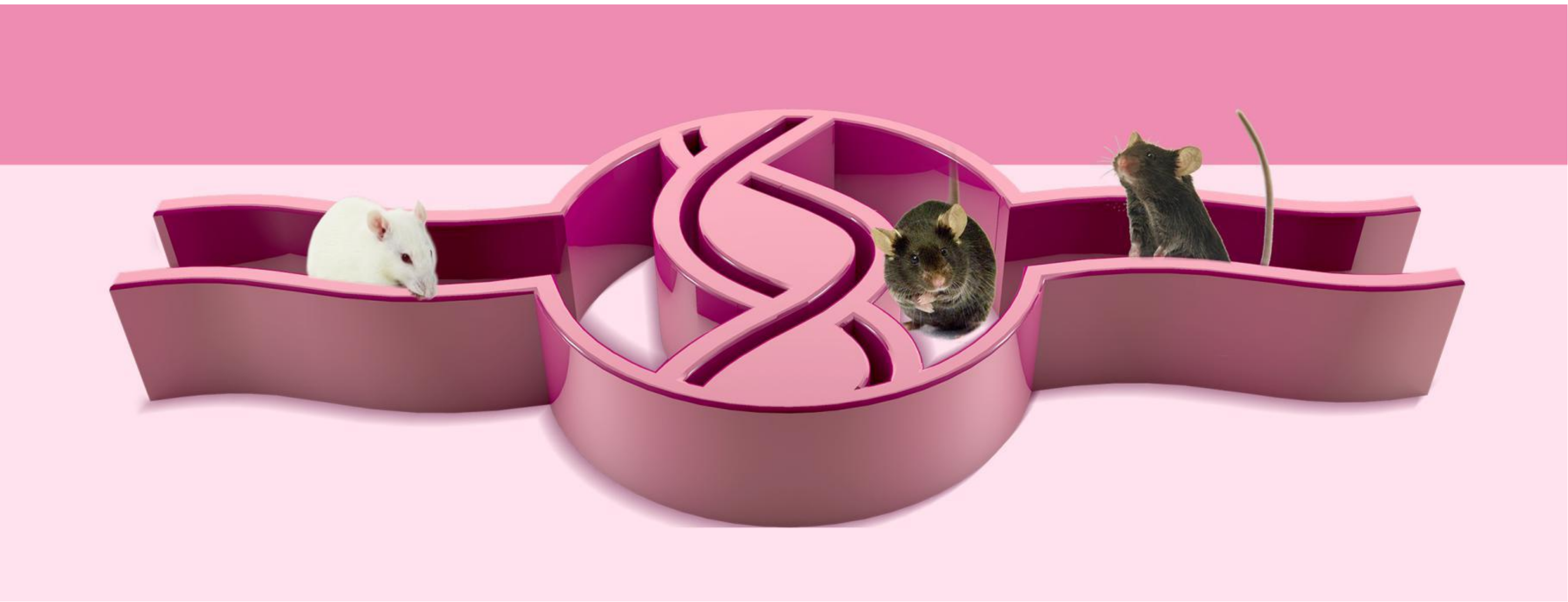

Mouse Conditional Knockout User Manual

Quote: CKOAI190919YZ7-B

- Confidential -

1. Product Information

Name	C57BL/6N-Nr1h4[em1cyagen]
Serial Number	CKOCMP-20186-Nr1h4-B6N-VA
Gene	Nr1h4
NCBI ID	20186
Strain	C57BL/6N
Type	conditional knockout

2. Targeting Strategy

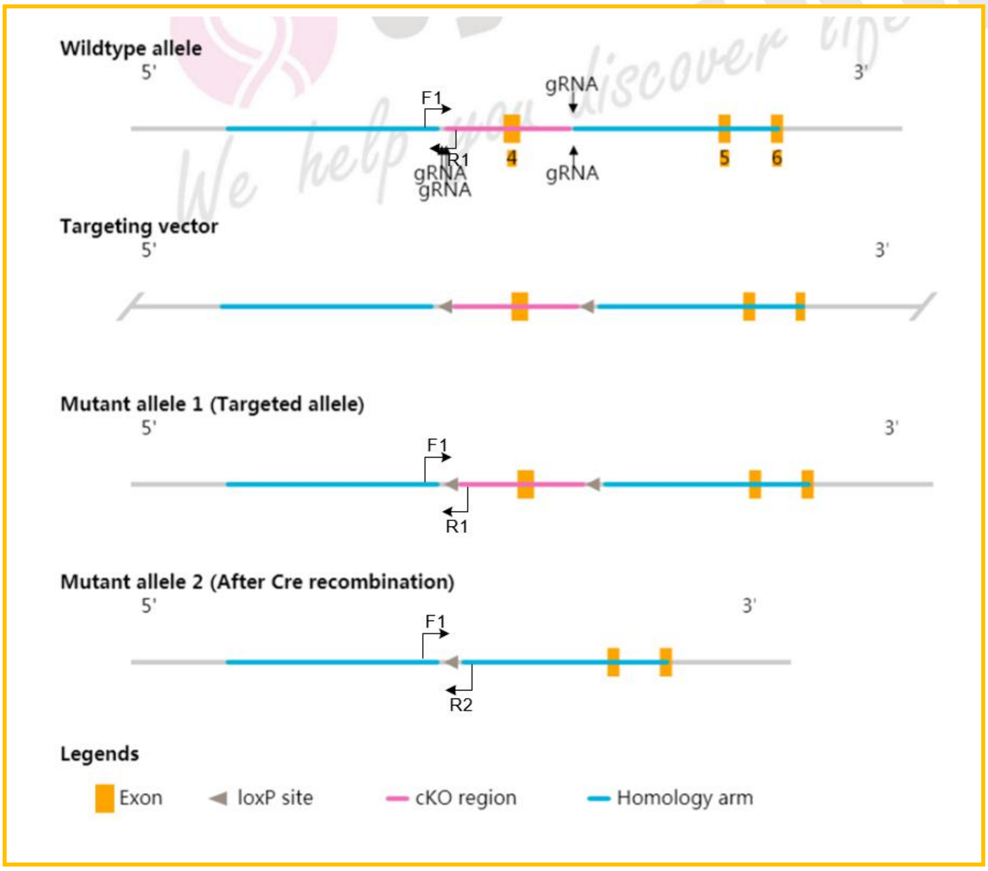

3. Genotyping strategy

<u>Primers1:</u> (Annealing Temperature 60.0 ºC)

F1: 5’-GTATACAGGATACCCAAGGAGGC-3’
R1: 5’-TGGCATTTAAAAGGAGAAGGCCAG-3’

Homozygotes: one band with 228 bp
Heterozygotes: two bands with 228 bp and 197 bp
Wildtype allele: one band with 197 bp

<u>Primers2:</u> (Annealing Temperature 60.0 ºC)

PVillin-M-F : 5’-GTGTTTGGTTTGGTTTCCTCTGCATAAGA-3’
PVillin-M-R : 5’-GCAGGCAAATTTTGGTGTACGGTCA-3’

Cre amplicon: ~500 bp

The tissue-specific gene deletion can be confirmed by adding one additional primer to the PCR assay:

<u>Primers4:</u> (Annealing Temperature 60.0 ºC)

F1: 5’-GTATACAGGATACCCAAGGAGGC-3’
R2: 5’-CTTTCCTTCTTAAGGGCAAGAGAG-3’

With Cre activity: one band with 230 bp

4. Expected Results

Genotyping	Primers1	Primers2
Flox/Flox, PVillin-Cre: Homozygotes, Cre	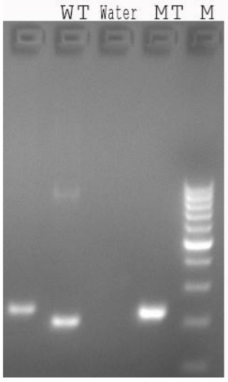	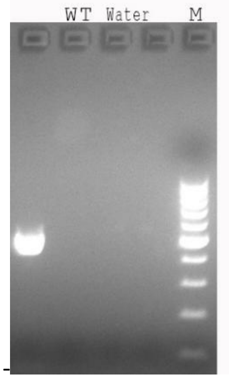

Note:

1) PCR was carried out in 25 µL volume for 35 cycles under standard conditions, with all two primers listed above added to each reaction.

cyagen We help you discover life

2) DNAmarker:Thermo Scientific GeneRuler 100 bp DNA Ladder #SM0242

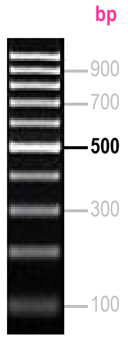

3) Controls used in PCR genotyping are:

- Water control: No DNA template added.
- Wildtype control: Mouse genomic DNA.
- Mutation control: Donor vector DNA.

cyagen We help you discover life

5. PCR reaction

5.1 DNA Extraction

➢ Method One:

We recommend that using TaKaRaMiniBEST Universal Genomic DNA Extraction kit (Ver.5.0_Code No. 9765) to gain high purity of genomic DNA.

a. Add 180 μL of Buffer GL, 20 μL of Proteinase K and 10 μL of RNase A per tail piece (2-5 mm) in a microcentrifuge tube. Be careful not to cut too much tail.

b. Incubate the tube at 56℃ overnight.

c. Spin in microcentrifuge at 12,000 rpm for 2 minutes to remove impurities.

d. Add 200 μL Buffer GB and 200 μL absolute ethyl alcohol with sufficient mixing.

e. Place the spin Column in a collection tube. Apply the sample to the spin and centrifuge at 12,000 rpm for 2 min. Discard flow-through.

f. Add 500 μL Buffer WA to the spin column and centrifuge at 12,000 rpm for 1 min. Discard flow-through.

g. Add 700 μL Buffer WB to the spin column and centrifuge at 12,000 rpm for 1 min. Discard flow-through. (Note: Make sure the Buffer WB has been premixed with 100% ethanol. When adding Buffer WB, add to the tube wall to wash off the residual salt.)

h. Repeat step g.

i. Place the spin Column in a collection tube and centrifuge at 12,000 rpm for 2 min.

j. Place the spin Column in a new 1.5ml tube. Add 50~200 μL sterilized water or elution buffer to the center of the column membrane and let the column stand 5min. (Note: Heating sterilized water or elution buffer up to 65℃ can increase the yield of elution.)

k. To elute DNA, centrifuge the column at 12,000 rpm for 2 min. To increase the yield of DNA, add the flow-through and/or 50~200 μL sterilized water or elution buffer to the center of the spin column membrane and let the column stand 5 min. Centrifuge at 12,000 rpm for 2 min.

l. Quantify to genomic DNA. Eluted genomic DNA can be quantified by electrophoresis or electrophoresis.

➢ Method Two:

A low-cost and sample method to gain rough genomic DNA.

a. Add 100 μL of tail digestion buffer per tail piece (2-5 mm) in a microcentrifuge tube. Be careful not to cut too much tail.

b. Incubate the tube at 56℃ overnight.

c. Incubate the tube at 98℃ for 13 minutes to denature the Proteinase K.

d. Spin in microcentrifuge at top speed for 15 minutes. Use an aliquot of supernatant straight from the tube (1 μL in a 12.5 μL reaction) for PCR.

Final concentration of tail digestion buffer:

➢ 50 mMKCl
➢ 10 mM Tris-HCl (pH 9.0)

- 0.1 % Triton X-100
- 0.4 mg/mL Proteinase K

5.2 PCR Mixture (primer concentration: 10μM):

Component	x1	
ddH_2O	9.0	μl
Product primer F	1.0	μl
Product primer R	1.0	μl
Premix Taq	12.5	μl
DNA	1.5	μl
Total	25	μl

5.3 PCR Reaction Conditions:

Step	Temp.		Time		Cycles
Initial denaturation	94	°C	3	min	
Denaturation	94	°C	30	s	35 x
Annealing	60	°C	35	s	
Extension	72	°C	35	s	
Additional extension	72	°C	5	min	

5.4 Relevant Reagents:

**TrizmaHydrochloride Solution**	Sigma, Cat. No. T2663
**Proteinase K**	Merck, Cat. No. MK539480
**Triton X-100**	Sigma, T8787-50 mL
**2 × Taq Master Mix (Dye Plus)**	Vazyme, P222
**Agarose**	BIOWEST AGAROSE, REGULAR
**DNA Marker**	Thermo Scientific GeneRuler 100 bp DNA Ladder #SM0242
**0.5×TBE**	Tris Bio Basic Inc, TBO194-500g
	EDTA Shanghai Sangon, 0105-500g
	Boric Acid, Shanghai Sangon, 0588-500g
