# Supplementary material for: FXR Maintains the Intestinal Barrier and Stemness by Regulating CYP11A1-Mediated Corticosterone Synthesis in Biliary Obstruction Diseases
Source: Int J Mol Sci. 2023 Aug 30;24(17):13494. doi: 10.3390/ijms241713494 (PMC10487515; doi:10.3390/ijms241713494)
Supplement: Supplementary file 1 [file ijms-24-13494-s001.zip › Supplemental information.pdf]

Supplemental Information

FXR Maintains the Intestinal Barrier and Stemness by Regulating CYP11A1-Mediated Corticosterone Synthesis in Biliary Obstruction Diseases

Zequn Li [1,2,3,4,†], Haijiang Dong [1,2,3,4,†], Suchen Bian [1,2,3,4], Hao Wu [1,2,3,4], Wenfeng Song [1,2,3,4], Xing Jia [1,2,3,4], Jian Chen [1,2,3,4], Xingxin Zhu [1,2,3,4], Long Zhao [1,2,3,4], Zefeng Xuan [1,2,3,4], Cheng Jin [1,2,3,4], Mengqiao Zhou [1,2,3,4], Shusen Zheng [1,2,3,4] and Penghong Song [1,2,3,4,*]

1 Division of Hepatobiliary and Pancreatic Surgery, Department of Surgery, The First Affiliated Hospital, Zhejiang University School of Medicine, Hangzhou 310003, China; 21718112@zju.edu.cn (Z.L.)
2 NHC Key Laboratory of Combined Multi-Organ Transplantation, Hangzhou 310003, China
3 Key Laboratory of the Diagnosis and Treatment of Organ Transplantation, Research Unit of Collaborative Diagnosis and Treatment for Hepatobiliary and Pancreatic Cancer, Chinese Academy of Medical Sciences (2019RU019), Hangzhou 310003, China
4 Key Laboratory of Organ Transplantation, Research Center for Diagnosis and Treatment of Hepatobiliary Diseases, Hangzhou 310003, China
* Correspondence: songpenghong@zju.edu.cn; Tel./Fax: +86-571-87236466.
† These authors contributed equally to this work.

Supplemental Materials and Methods

ELISA assay

Mice serum and intestine tissue homogenate supernatants were collected. And standard ELISA assay was performed using the Mouse Corticosterone (CORT) ELISA Kit (Keshun Science and Technology, Shanghai, China) according to manufacture instructions.

Cell transfection

The lentiviral vectors (hU6-MCS-Ubiquitin-EGFP-IRES-puromycin) containing small hairpin RNA (shRNA) targeting NR1H4 (LV-shFXR) or a non-target control (LV-Control) were purchased from Genechem. (Shanghai, China). And the sequences of shRNA were listed in Table S1. 200,000 Caco-2 cells per well were seeded in 6-well plates and infected by 2,000,000 UT LV-shFXR or LV-Control (Caco-2 MOI value=10) with using polybrene (Genechem., Shanghai, China). Successfully transfected Caco-2 cells were selected by a final concentration of 10 μg/mL Puromycin (MedChemExpress).

RNA isolation, qRT-PCR and RNA-seq

Total RNA of tissues and Caco-2 cells was extracted by using NucleoZOL (Macherey-Nagel, Düren, Germany). HiScript II Q RT SuperMix for qPCR (+gDNA wiper) Kit (Vazyme, Nanjing, China) was used for RNA reverse transcription. Then ChamQ Universal SYBR qPCR Master Mix (Vazyme,

Nanjing, China) and QuantStjudio 5 (Applied Biosystems, USA) was used to perform qRT-PCR. The primers sequences for qRT-PCR were listed in Table S1. The relative expression levels were normalized to Actb and expressed as $2^{-\Delta\Delta Cq}$. And RNA-Seq and transcriptome analysis was supported by Guangzhou Genedenovo Biotechnology Co., Ltd.

Western Blot analysis

The protein of tissues and Caco-2 cells was lysed using RIPA buffer (Thermo Fisher Scientific, USA) supplemented with Protease & Phosphatase inhibitor Cocktail (1:100; Thermo Fisher Scientific, USA) for 30 min on ice. Then protein samples were quantified using BCA Protein Assay Kit (Thermo Fisher Scientific, USA), and separated in SurePAGE™ gels 4-20% (GenScript, Nanjing, China), and transferred onto PVDF membranes (Millipore, USA) using eBlot™ L1 High Quality Wet Protein Transfer (GenScript, Nanjing, China). Membranes were blocked by 5% skim milk at room temperature for 1h, and incubated with primary antibodies at 4˚C overnight. The primary antibodies used for Western blot were listed in Table S2. The next day, membranes were incubated with secondary antibody at room temperature for 1h. TBS-T was used to wash PVDF membranes between the incubation gap. Finally, detected the signal on the membrane using Enhanced chemiluminescence reagent (Fdbio Science, Hangzhou, China) and Chemiluminescence Imaging System (Clinx Science Instruments, Shanghai, China).

Hematoxylin and eosin (HE) staining

Paraffin-embedded mice intestine tissues were cut into 4-μm slices, deparaffinized and rehydrated by dimethylbenzene and ethanol. Then hematoxylin was used to stain nucleus. And slides were soaking in the eosin dye for 1 min, running water flushing for 1min, gradually dehydrated by 75%, 85%, 95% ethanol and dimethylbenzene. Finally, slides were mounted with neutral resin (Zsbio, Beijing, China).

Sirius red staining

The slides were deparaffinized and rehydrated by dimethylbenzene and ethanol. Then slides were soaking in the Sirius red dye (Servicebio, Wuhan, China) for 15 min, running water flushing for 1min, gradually dehydrated by 75%, 85%, 95% ethanol and dimethylbenzene. Finally, slides were mounted with neutral resin.

Immunohistochemistry (IHC) and Immunofluorescence (IF) staining

The slides were deparaffinized and rehydrated by dimethylbenzene and ethanol. Then sections were soaking in 3% H2O2 for 10min at room temperature, and incubated with antigen repair buffer (pH 6.0 citrate buffer or pH 9.0 EDTA buffer) for antigen retrieval in microwave oven. Next, sections were blocked in 10% goat serum, and incubated with primary antibody

overnight at 4°C. For IHC, sections were incubated with a secondary antibody (Zsbio, Beijing, China) for 30min at 37°C, and stained with the 3'-diaminobenzidine (DAB), stopped by running water and sections were counterstained using hematoxylin. The steps of dehydration were same as HE staining. For IF, sections were incubated with a fluorescent secondary antibody (Thermo Fisher Scientific, USA) at room temperature for 1h (protected from light), then mounted by fluorescent mounting medium with DAPI (Zsbio, Beijing, China).

Intestinal permeability measuring

Mice were fasted for 8h, and gavaged with 4kD FITC-Dextran (Sigma-Aldrich, USA) at a dose of 500mg/kg. Portal vein blood of mice was collected and centrifuged after 4h. Then, 0, 6.25μg/mL, 12.5μg/mL, 25μg/mL, 50μg/mL, 100μg/mL and 200μg/mL 4kD FITC-Dextran solution was configurated. And the 488 fluorescence of serum samples and these 4kD FITC-Dextran solution was measured by Multifunction Microplate Reader (Thermo Fisher Scientific, USA).

Table S1. Primers sequences and shRNA sequences.

Name	Sequence (5′to 3′)
Actb	Forward: GTCATCACTATTGGCAACGAG
	Reverse: TGTGTTGGCATAGAGGTCTTTA
Ocln	Forward: TGGCAAGCGATCATACCCAGAG
	Reverse: CTGCCTGAAGTCATCCACACTC
Cldn1	Forward: GCTGTCATTGGGGGCATAA
	Reverse: GGAGCAGGAAAGTAGGACACC
Tjp1	Forward: GTTGGTACGGTGCCCTGAAAGA
	Reverse: GCTGACAGGTAGGACAGACGAT
Lgr5	Forward: CACAGCCTGGAGACTTTAGAT
	Reverse: GTATTGACCTGATGTTGTTGCT
Olfm4	Forward: CGAGAGAGAGTTTTCTAAGGTG
	Reverse: TCAAAGTCCAGTTCTGTGTAAG
Fxr (Nr1h4)	Forward: CTAATGAGGACGACAGCGAAG
	Reverse: CTGTTGGTCTGCCGTGAGTT
Tgr5	Forward: CACTGCTCTTCTTGCTGTGTTGG
(Gpbar1)	Reverse: GAGCGATAACAGAGTTCCAGGC
Fgf15	Forward: GAGGACCAAAACGAACGAAAT
	Reverse: GAGTAGCGAATCAGCCCGTAT
Cyp11a1	Forward: GCACACAGAAAATCCATTACCA
	Reverse: GAGAAGAGTATCGACGCATCCT
Agpat9	Forward: CGCTATTGCTTCCTGCTACCT
	Reverse: CAGATAGGGACCGAACACAGAT
Asah2	Forward: CCTTCTCACCCTCTTGTTTGTT
	Reverse: GTAGCCACTGAAGTTCCGAAAT
Acacb	Forward: GGAGGCAACAGGGTCATAGA
	Reverse: TTGATGTACTCTGCGTTGGC
Dgki	Forward: CAGGAGGGAAAATGTAAGCAG
	Reverse: GAGCAGGGTTCCTCAATATGA
Tlr2	Forward: ACAGCAAGGTCTTCCTGGTTCC
	Reverse: GCTCCCTTACAGGCTGAGTTCT
Tlr4	Forward: AGCTTCTCCAATTTTTCAGAACTTC
	Reverse: TGAGAGGTGGTGTAAGCCATGC

Cd14	Forward: TTGAACCTCCGCAACGTGTCGT
	Reverse: CGCAGGAAAAGTTGAGCGAGTG
Il1a	Forward: ACGGCTGAGTTTCAGTGAGACC
	Reverse: CACTCTGGTAGGTGTAAGGTGC
Il1b	Forward: TGGACCTTCCAGGATGAGGACA
	Reverse: GTTCATCTCGGAGCCTGTAGTG
Il6	Forward: CTAATGAGGACGACAGCGAAG
	Reverse: CTGTTGGTCTGCCGTGAGTT
Il10	Forward: CGGGAAGACAATAACTGCACCC
	Reverse: CGGTTAGCAGTATGTTGTCCAGC
Tnf (Tnf-α)	Forward: GGTGCCTATGTCTCAGCCTCTT
	Reverse: GCCATAGAACTGATGAGAGGGAG
shFXR	1- CTGACTGAATTACGGACAT
	2- ACTTCTTGATGTGCTACAA
	3- CTCAGGAAATAACAAATAA

Table S2. Antibodies.

Name	Catalog Number	Manufaturer
PCNA	ab92552	Abcam
TGR5	ab72608	Abcam
FXR	ab85606	Abcam
β-Actin	ab8226	Abcam
CYP11A1	ab272494	Abcam
Claudin 1	ab180158	Abcam
C-MYC	ab32072	Abcam
Lysozyme	ab108508	Abcam
OLFM4	39141	Cell Signaling Technology
ZO-1	61-7300	Thermo Fisher Scientific
MUC4	sc-33654	Santa Cruz

Supplementary Figures and Legends

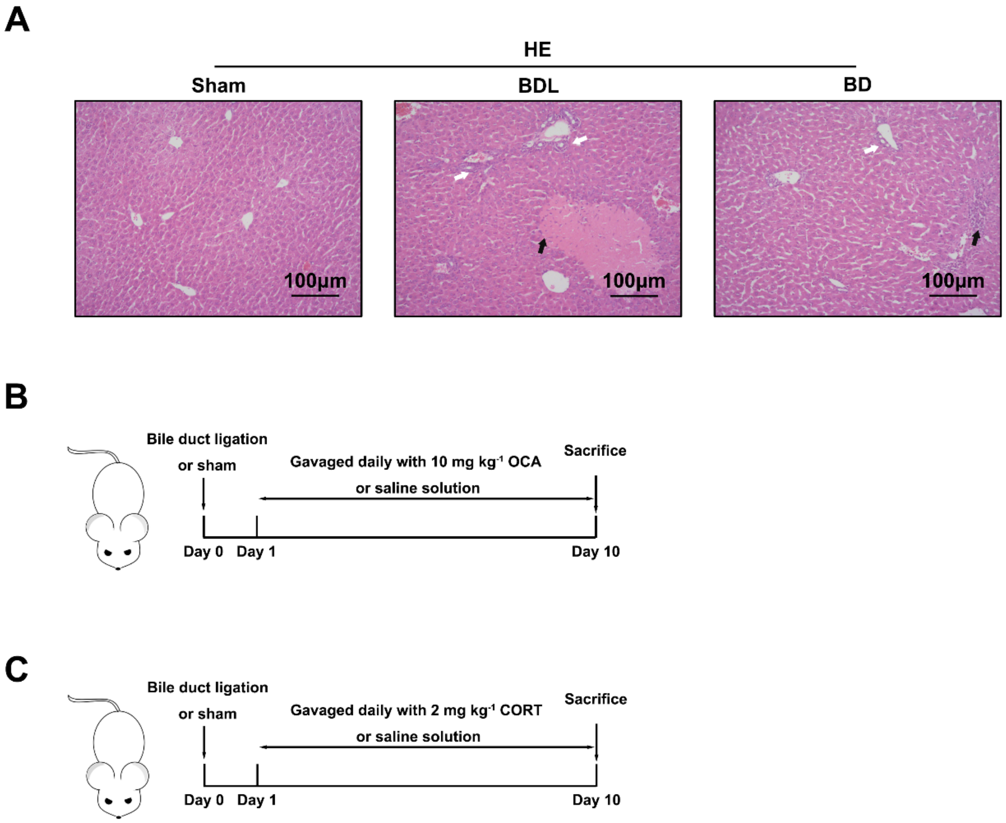

Figure S1. Intestinal bile acid deficiency induces bile duct hyperplasia and liver inflammation.

(A) HE staining of the liver in the sham, BDL and BD mouse models at 100× magnification. The white arrow indicates bile duct hyperplasia, and the black arrow indicates focal necrosis or inflammation.

(B) Schematic representation of sham or BDL mice gavaged with OCA or saline solution.

(C) Schematic representation of sham or BDL mice gavaged with CORT or saline solution.

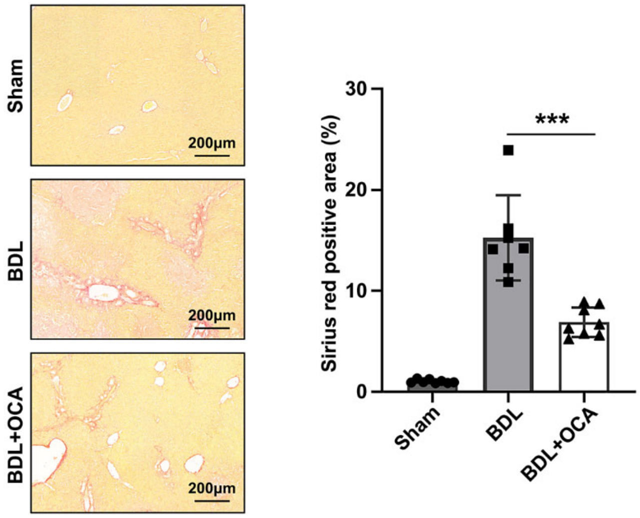

Figure S2. Activation of FXR decreases BDL mice liver injury.

Sirius Red S staining of the liver in the sham, BDL and BDL+OCA mice models at 50× magnification. Data are shown as the mean ± SD from per group, and statistical analysis was performed by one-way ANOVA: *** $P < 0.005$.

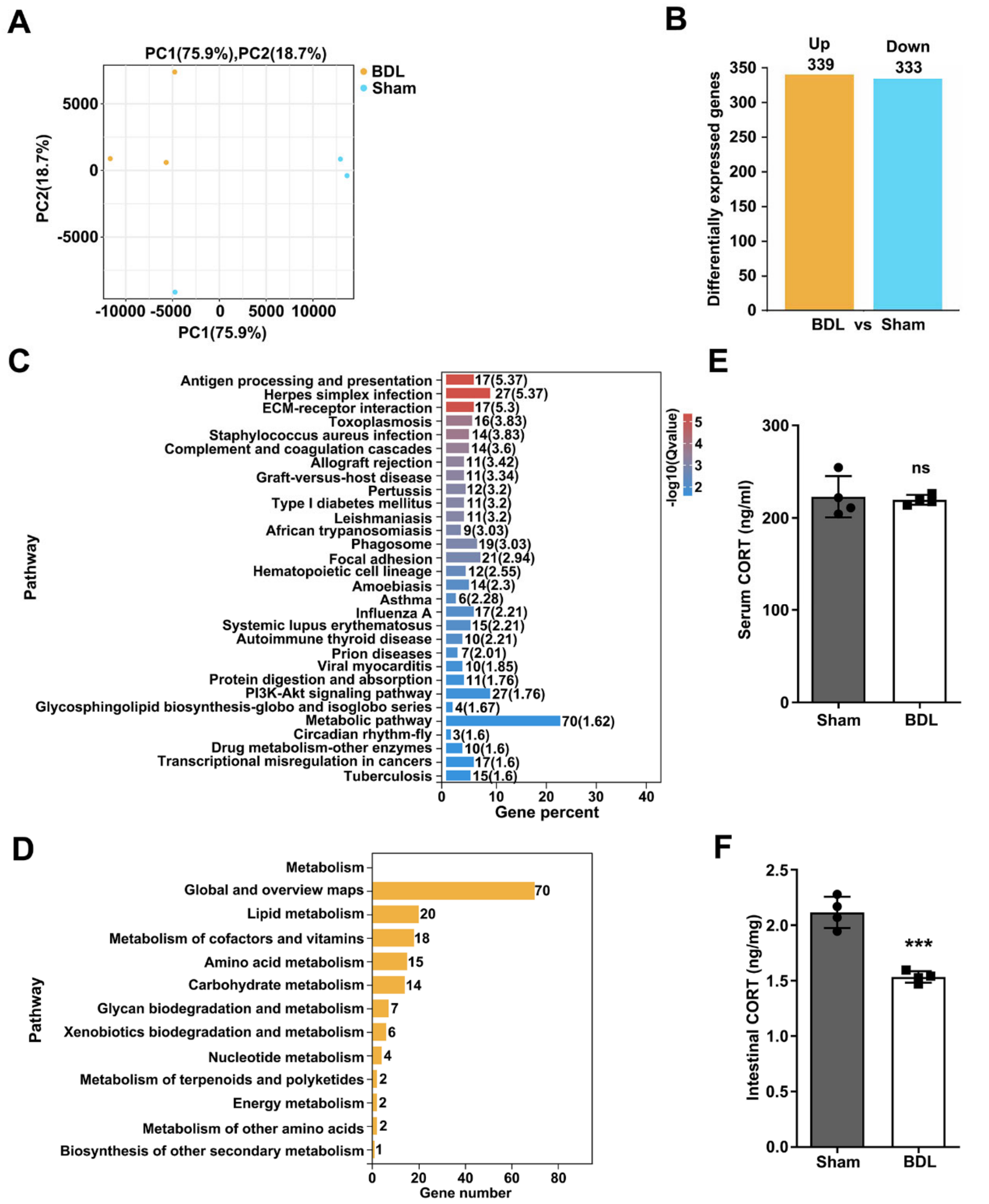

Figure S3. RNA-Seq analysis and CORT concentration difference between sham and BDL mice.

(A) Principal component analysis (PCA) between sham and BDL mouse crypt RNA-Seq, n=3 per group.

(B) Differential expression analyses used a 2.0-fold difference in expression.

(C) KEGG analysis of differentially expressed genes.

(D) KEGG analysis of metabolism-related genes among the differentially expressed genes.

(E) Serum CORT concentration of sham and BDL mice, n=4 per group. Data are shown as the mean ± SD from per group, and statistical analysis was performed by unpaired Student's t test.

(F) Intestinal CORT concentration of sham and BDL mice, n=4 per group. Data are shown as the mean ± SD from per group, and statistical analysis was performed by unpaired Student's t test: *** $P < 0.005$.

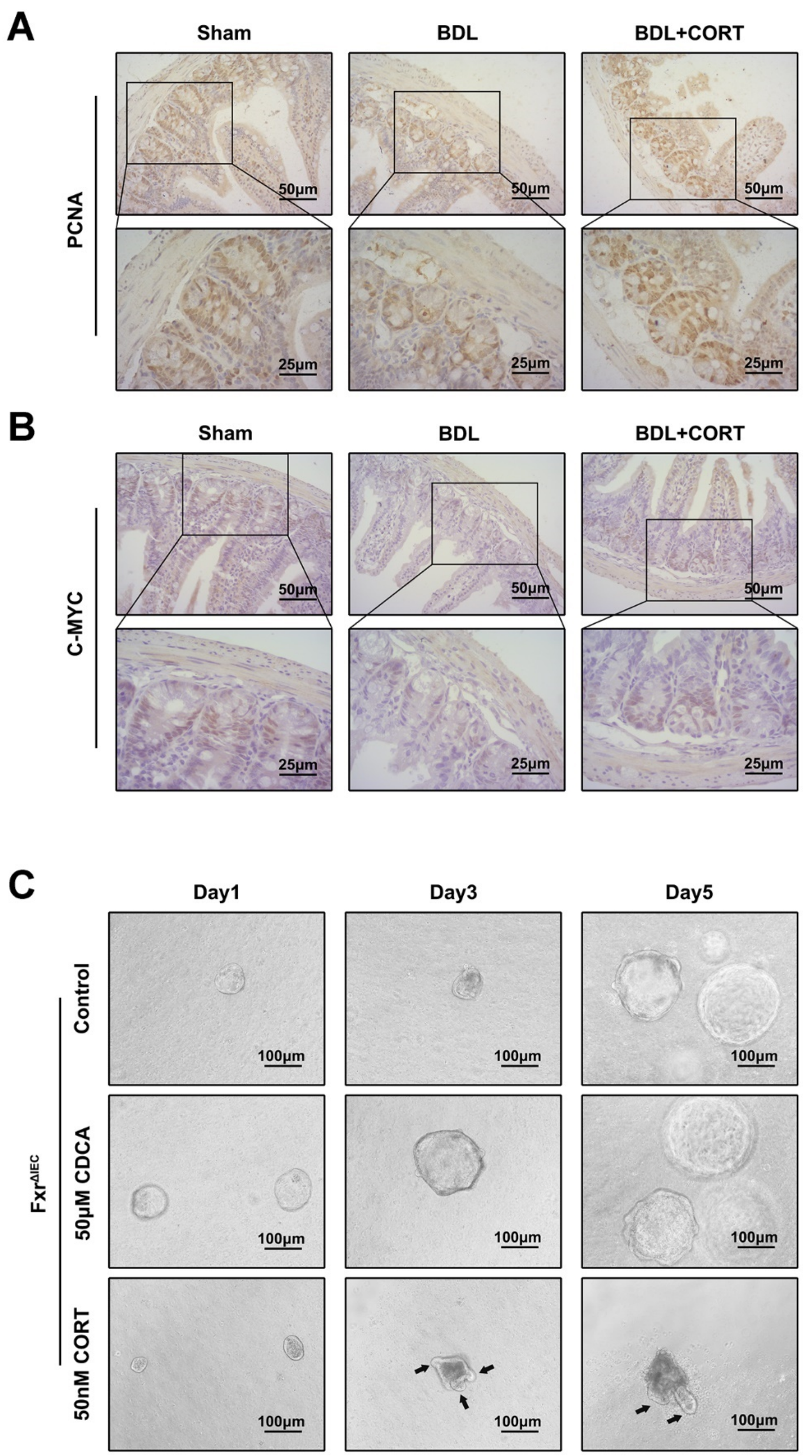

Figure S4. CORT promotes intestinal crypt cell proliferation and maintains intestinal stemness.

(A) Intestine PCNA IHC staining of sham, BDL and BDL+CORT mice; up

photos with a 200× magnification and down photos with a 400× magnification.

(B) Intestine C-MYC IHC staining of sham, BDL and BDL+CORT mice; up photos with a 200× magnification and down photos with a 400× magnification.

(C) $Fxr^{\Delta IEC}$ intestine organoids were cultured with 50 μM CDCA, 50 nM CORT, or DMSO as a control, and the development of organoids on days 1, 3 and 5 was recorded at 100× magnification. The black arrow indicates the newborn crypts.

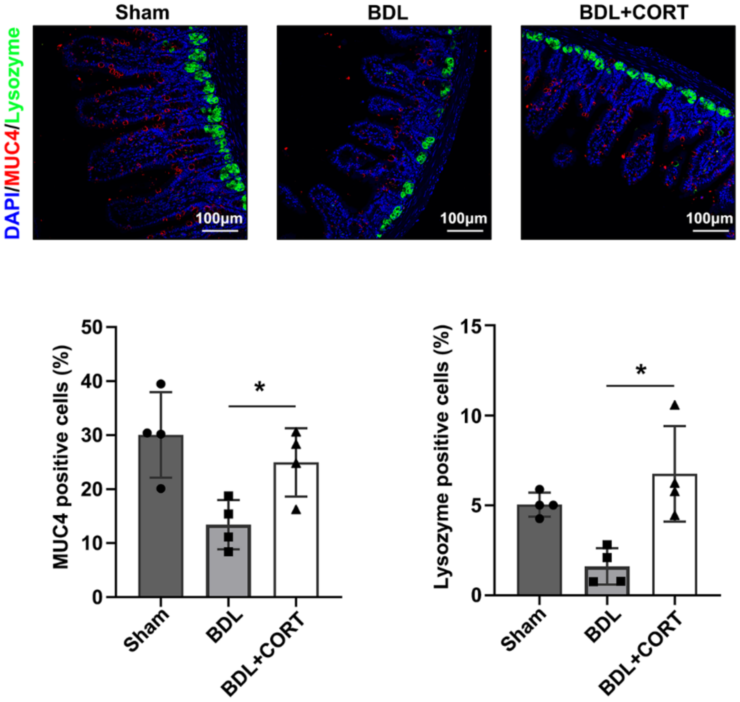

Figure S5. CORT attenuates the loss of goblet cells and Paneth cells in BDL mice. Immunofluorescence staining of sham, BDL and BDL+CORT mice intestines, DAPI for unclear labelling, MUC4 was red for goblet cell labelling, and lysozyme was green for Paneth cell labelling, with 200× magnification. Data are shown as the mean ± SD from per group, and statistical analysis was performed by one-way ANOVA: *** $P < 0.005$.
